# Psychological profiles, including emotion regulation characteristics, defence strategies and mentalisation capacity, of male adolescents involved in fights among peers

**DOI:** 10.1192/bjo.2022.634

**Published:** 2023-01-26

**Authors:** Silvia Cimino, Luca Cerniglia

**Affiliations:** Department of Dynamic and Clinical Psychology and Health Studies, Sapienza University of Rome, Italy; Faculty of Psychology, International Telematic University UniNettuno, Italy

**Keywords:** Youth violence, mental health and violence, community violence, adolescents, mentalising

## Abstract

**Background:**

Youth violence has become a worrying public health issue worldwide. In Europe and the USA, research has shown a prevalence of this phenomenon ranging from 30 to 70% in boys.

**Aims:**

This descriptive study aimed to evaluate psychological profiles of male adolescents involved in fights with their peers.

**Method:**

Identity consolidation was evaluated with the Self-Concept and Identity Measure; defence strategies were assessed by the Response Evaluation Measure for Youth; emotion regulation was assessed with the Difficulties in Emotion Regulation Scale and mentalisation capacity was evaluated by the Reflective Functioning Questionnaire.

**Results:**

Through a series of multivariate analyses of variance, our results showed that adolescents reporting four or more fights in the past year, when compared with peers reporting none or fewer than four fights, displayed lower identity consolidation, greater use of immature defence strategies, poorer emotion regulation processes and poorer mentalisation capacity.

**Conclusions:**

The results of this study could be useful for the promotion of prevention and intervention programmes to stem fights among adolescents.

Adolescence is a decisive period for the construction of adult identity and stabilisation of personality, and is accompanied by important transformations and processes of change on the body and cognitive and affective levels.^1,2^ However, adolescence can be considered an at-risk period, especially because it is usually marked by an increase in arousal related to the biological and physiological transformations of puberty, and brain development is generally incomplete in the prefrontal cortex, which is involved in the processes of metacognition, self-evaluation and self-regulation.^3,4^ For these reasons, it may be considered a developmental phase of increased vulnerability for the onset of affective and behavioural dysregulation, leading to internalising and externalising problems (e.g. conduct disorders^5^), and to difficulties in the construction and stabilisation of identity. It has been suggested that a consolidated identity refers to the perception of feeling whole, linked to the past and confident about oneself; conversely, a disturbed identity consist of difficulties such as identity confusion, fragmentation, uncertainty and discontinuity. Some individuals may also refer a sense of lacking an identity, feeling a void or being senseless.^6,7^ The characteristics of adolescents’ emotional and behavioural functioning, the detachment from the origin family and the importance of the peer group are posited to be typical of such a developmental phase and are useful as transition stages toward adulthood.^8^ However, in some cases adolescents can show intense maladaptive behaviours (e.g. alcohol and drug use, withdrawal, aggressiveness, frequent fights among peers).

## Violent behaviors in adolescence

In particular, research has shown that numbers of violent behaviours and fights among adolescents are continuously growing in Europe; in Italy, almost 20% of youths have been involved in interpersonal violence in the past year.^9–11^ Ten years ago, similar studies found a prevalence of 10–15%, accounting for a worrying 5–10% increase in this phenomenon. Research has suggested that a number of underpinning variables could be associated with these problems. Very importantly, an effective emotional and behavioural regulation process is suggested to be crucial to hamper maladaptive conducts,^12^ and an adaptive response to hyperarousal (which is typical and normal in adolescence) can be assisted by mature defence strategies.^13^ Defence strategies are unconscious reactions, not planned responses, and form an interface between base characteristics (e.g. impulsivity) and learned coping strategies.^14^ They may be conceptualised on a continuum than ranges from immature (e.g. Passive aggression, omnipotence, denial, acting out conversion, withdrawal) to mature strategies (e.g. repression, humour, sublimation, intellectualisation).^15^ Immature strategies alter reality in agreement with anticipated consequences, resulting in more maladaptive functioning. Mature defences attenuate unwelcome reality, allowing for more adaptive functioning.^16^

### Mentalisation capacity

Moreover, researchers have posited that an impaired mentalisation capacity can be associated with aggressiveness and rule-breaking.^17^ Understanding others and oneself in terms of internal mental states, such as beliefs, desires, feelings and attitudes, is defined as mentalisation.^18^ Affect regulation, the creation and maintenance of a strong sense of self, and fruitful social relationships are all made possible by high levels of mentalisation.^19^ Hypo-mentalisation and hyper-mentalisation are two ways that a deficit in this ability may manifest, according to Fonagy et al.^20^ Hypo-mentalisation is characterised by concrete thinking and a lack of understanding of one's own and other people's mental states, whereas hyper-mentalisation is characterised by an over-mentalising attitude that involves developing too specific and in-depth models of the mind and mental states that are not supported by the available data. In sum, adjusting and inhibiting emotional and/or behavioural capacities of adolescents are posited to be associated with key variables, such as consolidated identity, the nature of defence strategies (mature versus immature), adaptive/maladaptive emotion regulation and mentalisation capacity. We recognise, of course, the role of other crucial variables such as bio-physiological individual characteristics (e.g. dopamine, cortisol and opioid secretion and transmission), prosocial behaviour and/or moral disengagement processes. However, in this descriptive study, we chose to focus on psychodynamic variables with the specific aim of evaluating the possible differences in emotional and behavioural characteristics of two groups of male adolescents: a group that engage in frequent physical fights and a group that engage in occasional or no fights.^21^

## Method

### Sample and procedure

Thanks to the cooperation of public and private schools, we were able to gather a convenience sample from the general community (adolescents’ school grades varied from ninth to 12th grade). The following inclusion criteria were applied: the individuals did not have a psychiatric diagnosis and did not receive any medical or psychological treatment. We recruited 537 adolescents (age range: 14–17 years; all male). Youths who refused to participate in the study (*n* = 25), those whose parents refused to allow their children to participate (*n* = 27) and those with a psychiatric diagnosis (*n* = 2) were all excluded from the sample. As a result, the final sample size for this study was 483 male adolescents. The majority of the youths (96.8%) were White, and 71% of their households earned between €28 000 and €55 000 per year (average socioeconomic status). Among the adolescents, 82.2% belonged to intact family units.

The authors state that all procedures used in this study adhere to the ethical norms of the appropriate national and institutional human experimentation committees, as well as the Helsinki Declaration of 1975, as updated in 2008. The Ethical Committee of La Sapienza, University of Rome, authorised all procedures involving human patients before the initiation of the current investigation (approval number 0023/22). All parents or guardians of the participants completed a written informed consent document. Any personal information was assured to be anonymous and private. At school, under the supervision of a member of the research team and the teacher, the participants filled out a series of self-report measures and an *ad hoc* questionnaire prepared following the work of de Looze et al, asking about their sociodemographic information and one specific question about physical violence: ‘During the past 12 months, how many times were you in a physical fight?’. Response categories were never, once, twice, three times and four or more times. Based on the study by de Looze et al^21^, this variable was dichotomised to assess recurrent fighting behaviour, to identify young individuals for whom violence was likely habitual as opposed to an infrequent or non-existent conduct (0 = fewer than four times; 1 = four times or more). Based on their responses, adolescents were divided into two groups: group 1, including youths who declared fewer than four fights in the past year; and group 2, including those reporting four or more than four fights in the past year. Table 1 shows demographic characteristics of the groups.

**Table 1:** Demographic characteristics of individuals in group 1 (fewer than four fights in the past year) and group 2 (four or more fights in the past year)

	Group 1	Group 2	Significance
Participants, *n*	368	115	−
Age, years, mean (s.d.)	15.6 (0.87)	15.9 (1.01)	Not significant
Household income per year, mean	€31 000	€32 000	Not significant
Intact families	83%	85%	Not significant

Significance set at *P* > 0.05.

### Tools

To assess identity consolidation the Self-Concept and Identity Measure (SCIM^7^) was used. This tool is a 27-item self-report measure that assesses identity consolidation and clinically relevant identity disturbance. SCIM items were designed to examine core aspects of identity, including (a) self-concept and role continuity across environments and among different persons, (b) consistencies in values and interests, (c) self-worth, (d) self/other-differentiation and (e) cohesion (i.e. feeling whole or complete). Participants were asked to indicate how much they agree or disagree with 27 self-focused statements. Response options range from 1 to 7 (1 = strongly disagree; 7 = strongly agree). Total scores were created in which all items were recoded such that higher scores mark greater identity disturbance. Although no clinical cut-off values have been established in previous literature, a total score <60 has been correlated with non-clinical samples.^22^ In this study, the tool yielded high internal consistency (Cronbach's *α* = 0.88).

To evaluate defence strategies, the Response Evaluation Measure for Youth (REM-71) was used.^23,24^ This is a self-report questionnaire with 71 items that allows for the examination of 21 defensive systems (acting out, splitting, displacement, dissociation, fantasy, passive aggression, projection, repression, omnipotence, undoing, conversion, somatisation, withdrawal, suppression, denial, humour, intellectualisation, reaction formation, idealisation, altruism, sublimation). Each defence strategy is evaluated using three or four items, and graded on a nine-point scale ranging from ‘strongly disagree’ (1) to ‘strongly agree’ (9). Higher scores on this measure indicate a greater usage of defensive measures. The structure of this tool is two-factor. Factor 1 consists of maladaptive defences with a higher rate of reality distortion, which leads to less flexible functioning (defence systems 16 to 21 of the REM-71). A cut-off of >4.40 discriminates clinical from non-clinical populations. In this study, the tool yielded high internal consistency (Cronbach's *α* = 0.89), and the current Italian version, demonstrated a good internal consistency and test–retest reliability (reliability coefficient of 0.84).

To measure emotion regulation, the Difficulties in Emotion Regulation Scale (DERS) was used.^25^ The DERS is a 36-item self-report questionnaire that evaluates the six dimensions of emotion regulation: (a) lack of emotional awareness (e.g. ‘I am attentive to my feelings’ [reversed]); (b) lack of emotional clarity (e.g. ‘I have no idea how I am feeling’); (c) impulse control difficulties (e.g. ‘When I'm upset I feel out of control’); (d) difficulties in engaging in goal directed behaviours (e.g. ‘When I'm upset I have difficulties getting work done’); (e) non-acceptance of emotional responses (e.g. ‘When I'm upset I feel guilty for feeling that way’) and (f) limited access to emotion regulation strategies (e.g. ‘When I am upset my emotions feel overwhelming’). The items are rated in terms of how frequently each statement applies to them on a five-point Likert scale ranging from 1 (‘almost never’) to 5 (‘almost always’). The total score for the DERS is calculated by adding all of the answers, and the subscale scores are also added, for a total score (clinical cut-off >84). The DERS has demonstrated high internal consistency, with a Cronbach's *α* of 0.93. In the current study, the Cronbach's *α* was 0.94 for the total scale for adolescents, 0.76 for awareness, 0.81 for clarity, 0.85 for impulsivity, 0.86 for goals, 0.87 for non-acceptance and 0.86 for strategies.

To measure mentalisation capacity, the Reflective Functioning Questionnaire (RFQ) was used.^20^ The RFQ has two subscales evaluating levels of confidence and uncertainty regarding mental states, each with six items (RFQ_U and RFQ_C). Six questions make up the RFQ_U subscale, which measures participant agreement with statements such as ‘Sometimes I act without truly understanding why’. Participants rate items using a seven-point Likert scale, which ranges from ‘totally disagree’ to ‘absolutely agree’. Items are rescored to 0, 0, 0, 0, 1, 2 and 3, to capture extreme levels of uncertainty. As a result, very high agreement on this scale indicates hypo-mentalisation (i.e. a lack of knowledge about mental states), whereas some disagreement indicates an adaptive recognition of the opacity of one's own mental states, which is typical of genuine mentalisation. The RFQ_C subscale consists of six items such as ‘I don't always know why I do what I do’, and is scored on the same seven-point Likert scale as the RFQ_U, then rescored to capture extreme levels of certainty; to this end, these items are recoded as 3, 2, 1, 0, 0, 0 and 0. As a result, low agreement reflects hyper-mentalisation, whereas some agreement reflects adaptive levels of certainty about mental states. Although no clinical cut-off values have been established in previous literature, total scores of <1.50 on the RFQ_C subscale and <1.8 on the RFQ_U subscale have been associated with non-clinical samples.^20^ In this study, the tool yielded high internal consistency (Cronbach's *α* = 0.87).

### Analyses plan

We carried out a series of multivariate analyses of variance (MANOVAs) in group 1 and group 2 to evaluate characteristics of identity, the nature of defence strategies (mature versus immature), emotional and behavioural regulation capacity and mentalisation ability. A power analysis was conducted according to Cohen's^26^ suggestions, α was set at 0.05, and a power of 0.876 was obtained with a large effect size (*f*^2^ = 0.46). All analyses were performed with SPSS software version 21.0 for Windows.

## Results

### Characteristics of identity

Results of the MANOVA showed that there was a statistically significant difference between the two groups in the dimension of Identity Consolidation (*P* > 0.01). In particular, Bonferroni's post-hoc test showed that Group 2 (adolescents reporting four or more fights in the past year) scored significantly lower Identity Consolidation. No other significant differences were instead found on the other sub-variables of Identity Disturbance and Lack of Identity. Table 2 shows eta-square coefficients for all the sub-scales. Considering the total scores, Group 1 did not exceed scores >60 associated with clinical samples, whereas Group 2 did.

**Table 2:** Mean scores for group 1 (fewer than four fights in the past year) and group 2 (four or more fights in the past year) on the Self-Concept and Identity Measure

	Group 1	Group 2	*η*^2^	*F*
	Mean (s.d.)	Mean (s.d.)		
Identity consolidation	20.88 (1.31)	44.01 (1.42)	0.67*	26.51_(__1.481)_
Identity disturbance	18.85 (1.01)	20.35 (1.21)	0.21	11.54_(__1.481)_
Lack of identity	22.74 (1.25)	24.57 (1.34)	0.19	12.36_(__1.481)_

* *P* > 0.01.

### Use of mature versus immature defence strategies

Results of the MANOVA showed that there was a statistically significant difference between the two groups in use of immature defences (*P* > 0.01). In particular, Bonferroni's *post hoc* test showed that group 2 (adolescents reporting four or more fights in the past year) scored significantly higher on immature defences and lower on mature defences. Moreover, participants in group 2 showed significantly higher scores in the dimensions of acting out, splitting, displacement and omnipotence. Conversely, participants in group 2 showed significantly lower scores in the dimension of withdrawal. Further, adolescents reporting four or more than four fights in the past year (group 2) showed significantly lower scores in the dimensions of humour and intellectualisation. Group 1 did not exceed the cut-off (>4.40) on the immature defences scale, whereas group 2 did. Table 3 shows *η*^2^ coefficients for all of the subscales.

**Table 3:** Mean scores and for group 1 (fewer than four fights in the past year) and group 2 (four or more fights in the past year) on the Response Evaluation Measure for Youth

	Group 1	Group 2	*η*^2^	*F*
	Mean (s.d.)	Mean (s.d.)		
Immature defences	4.62 (3.64)	8.22 (2.78)*	0.68*	34.21_(__1.481)_
Acting out	3.82 (1.24)	5.34 (1.21)	0.69*	32.52_(__1.481)_
Splitting	6.23 (4.21)	7.46 (5.56)	0.86*	34.32_(__1.481)_
Displacement	4.89 (1.51)	6.08 (1.67)	0.67*	28.26_(__1.481)_
Dissociation	6.24 (1.52)	5.98 (1.11)	0.22	10.64_(__1.481)_
Fantasy	6.14 (3.74)	5.89 (4.41)	0.13	8.47_(__1.481)_
Omnipotence	5.61 (2.71)	7.65 (1.64)	0.71*	29.45_(__1.481)_
Passive aggression	4.35 (1.61)	4.49 (2.43)	0.21	10.58_(__1.481)_
Projection	5.51 (1.44)	5.74 (1.42)	0.14	9.74_(__1.481)_
Repression	5.42 (1.25)	5.14 (1.87)	0.24	11.71_(__1.481)_
Undoing	5.84 (1.33)	6.05 (1.46)	0.17	8.36_(__1.481)_
Sublimation	6.14 (1.22)	4.17 (1.58)	0.25	9.75_(__1.481)_
Conversion	3.24 (1.65)	2.56 (1.74)	0.19	9.56_(__1.481)_
Somatisation	4.47 (1.91)	4.89 (1.56)	0.21	9.41_(__1.481)_
Withdrawal	6.58 (1.29)	5.32 (3.25)	0.71*	31.22_(__1.481)_
Mature defences	6.32 (1.54)	4.52 (1.54)	0.79*	32.41_(__1.481)_
Altruism	6.81 (1.13)	7.42 (1.22)	0.20	12.01_(__1.481)_
Denial	5.72 (2.15)	5.61 (2.78)	0.12	7.25_(__1.481)_
Humour	7.14 (1.54)	5.32 (1.18)	0.78*	35.05_(__1.481)_
Idealisation	6.35 (1.61)	6.87 (1.56)	0.22	10.46_(__1.481)_
Intellectualisation	7.41 (2.93)	5.37 (2.19)	0.81*	35.61_(__1.481)_
Reaction formation	4.74 (2.81)	4.39 (2.67)	0.13	9.44_(__1.481)_
Suppression	4.47 (1.83)	4.83 (2.89)	0.21	7.33_(__1.481)_

* *P* > 0.01.

### Emotional and behavioural regulation

Results of the MANOVA showed that there was a statistically significant difference between the two groups in the total scores, impulse control difficulties and lack of emotional clarity (*P* > 0.01). In particular. Bonferroni's *post hoc* test showed that group 2 scored significantly higher on all of the above dimensions. Table 4 shows *η*^2^ coefficients for all of the subscales. Considering the total scores, group 1 did not exceed the clinical cut-off (>84), whereas group 2 did.

**Table 4.** Mean scores and for group 1 (fewer than four fights in the past year) and group 2 (four or more fights in the past year) on the Difficulties in Emotion Regulation Scale

	Group 1	Group 2	*η*^2^	*F*
	Mean (s.d.)	Mean (s.d.)		
Impulse control difficulties	11.83 (4.22)	13.24 (3.51)	0.73*	35.05_(__1.481)_
Difficulties in goal-directed behaviour	14.75 (4.81)	14.86 (3.61)	0.21	10.06_(__1.481)_
Non-acceptance of emotional response	14.32 (5.01)	14.44 (4.82)	0.19	8.54_(__1.481)_
Lack of emotional awareness	14.55 (4.35)	14.98 (4.31)	0.22	9.76_(__1.481)_
Limited access to emotion Regulation strategies	16.32 (5.21)	19.45 (4.41)	0.18	9.88_(__1.481)_
Lack of emotional clarity	11.21 (2.98)	14.42 (4.24)	0.81*	36.32_(__1.481)_
Total	82.98 (2.01)	91.36 (3.23)	0.74*	32.83_(__1.481)_

* *P* > 0.01.

### Mentalisation capacity

Results of the MANOVA showed that there was a statistically significant difference between the two groups on the RFQ_C and RFQ_U (*P* > 0.01). In particular, Bonferroni's *post hoc* test showed that group 2 scored significantly higher on both of the above dimensions. Considering the total scores, group 1 did not exceed scores associated with clinical samples, whereas group 2 did. Table 5 shows *η*^2^ coefficients for all of the subscales.

**Table 5:** Mean scores for group 1 (fewer than four fights in the past year) and group 2 (four or more fights in the past year) on the Reflective Functioning Questionnaire

	Group 1	Group 2	*η*^2^	*F*
	Mean (s.d.)	Mean (s.d.)		
RFQ Certainty of mental states	1.41 (1.18)	2.85 (1.19)	0.72*	32.65_(__1.481)_
RFQ Uncertainty of mental states	1.65 (1.12)	5.21 (1.43)	0.79*	34.21_(__1.481)_

* *P* > 0.01.

### Discussion

Youth violence has become a worrying public health issue worldwide. In Europe and the USA, research has shown a prevalence of this phenomenon ranging from 30 to 70% in boys.^27^ Given the amplitude and the severity of this question, the World Health Organization has urged to act and implement prevention and intervention policies. However, in most cases, plans to reduce or prevent fights among adolescents are being based on information campaigns with the goal of sensitising and educating youth to the dangers and effects of such behaviours.^28^ The effectiveness of these programmes, nevertheless, is highly impaired by the fact that no or limited attention is given to specific characteristics of emotional or behavioural functioning of these youths, so that no specific port of entry for intervention and prevention is proposed. There is a need, therefore, for studies assessing dimensions that have been generally associated with aggressiveness but have not been sufficiently considered when focusing on fights among adolescents.

This descriptive study aimed at filling this gap by assessing the functioning of a number of psychodynamic variables in two groups of adolescents recruited from the general population (group 1 reporting no or occasional involvement in fights and group 2 reporting four or more than four fights in the past year), verifying possible differences between them.

The results of this study showed that adolescents who reported four or more than four fights in the past year (group 2) scored significantly higher than their peers with no or fewer than four fights, on the use of immature defence strategies. In particular, they reported to heavily use acting out, splitting, displacement and omnipotence. Youths in group 2 also showed significantly lower scores in the dimensions of humour and intellectualisation, which are mature defence strategies. Recent research has demonstrated that the use of immature defence strategies in adolescents is associated with difficulties in their emotional and/or behavioural regulation processes.^29^ Considering that the DSM-5 defines defences as mechanisms that mediate the individual's reaction to emotional conflicts and external stressors, it can be posited that immature strategies are maladaptive and ineffective tools through which the mind unconsciously attempts to modulate the distress experienced in its relationship with the environment and other individuals.^30^ In this sense, immature defence strategies could be considered either predictors or outcomes of ineffective emotion regulation processes. Most importantly, the cluster of immature strategies cited above has been suggested as associated with borderline personality organisation.^31^ As it is widely known, individuals with this personality organisation are characterised by subtle shifting from idealisation to despising others, and compromised sense of reality when under stress.^32^ These characteristics could facilitate maladaptive strategies of conflict resolution, therefore accelerating the use of violence to compose divergences with others or even just cope with unpleasant emotions.

The unpleasantness of an emotion is often the result of the individual's difficulty in clearly distinguishing it.^33^ Adolescents in group 2 showed high lack of emotional clarity, together with impulse control difficulties. Hence, the violent behaviour could result from a combination of impulsivity and a sort of blindness toward affective states, leading to ineffective regulation strategies. This hypothesis is corroborated by another interesting result of this study that is the high scores of group 2 in the dimensions of certainty of mental states and uncertainty of mental states (of others). High scores on these dimensions account for an unrealistic representation of one's own reflective function capacity (either too high or two low), and can be interpreted as a individual's difficulty in grasping their own and others’ mental states and intentions. In one case (hyper-mentalisation), the individual feels that they can faultlessly understand mental states; in the other case, the individual feels that they are completely unable to understand mental states. Either way, the misinterpretation of one's own and others’ intentions and internal states has been widely associated with violent responses to stressful situations and impairment in resolving conflicts peacefully,^31^ which could be at the basis of adolescents’ frequent involvement in fights and violent behaviours.

Another noteworthy result of our study is that youths in group 2 displayed lower identity consolidation. Consolidating one's identity is one of the fundamental developmental skills that has been shown to be protective against these and other emerging behaviours that can compromise adult mental health.^19^ The main psychological work adolescents must accomplish is creating a sense of individual identity. According to Kernberg, youths lacking these characteristics may be the most inclined to engage in risky conduct, either in an effort to discover who they are or to relieve the tension brought on by identity conflicts.^33^ Our result is therefore consistent with previous literature in that adolescents with poorer identity consolidation were more frequently involved in fights among peers. Our study adds to previous studies because, to the best of our knowledge, identity consolidation has not previously been considered as a dimension associated with fights among youths.

The results of this study may offer useful information that might be used in clinical settings. In fact, the present research confirms the effectiveness of school-based identification of youths at risk for maladaptive psychological functioning associated with aggressive and violent behaviour.^34^ These aspects are frequently associated with suicide risk and severe depressive symptoms, and the assessment of such problems in the general population may inform prevention and intervention programmes involving schools, families and the community.^35^ Interventions might be directed in particular to the enhancement of adolescents’ mentalisation capacity, emotion regulation and the use of mature defence strategies, especially in situations that confront them with distress and conflict, with the aim of reducing the prospect of physical aggression and violence as a maladaptive response to poor regulation.^36^

This study has limitations. First, this research is not able to draw any causal conclusions from the gathered data because of the cross-sectional nature of its design. Second, this study was able to recruit only male adolescents. This was because the schools that agreed to be part of the research were attended almost entirely by males. This could constitute a bias and limits the generalisability of our results. Another limit to the generalisability of our results is that almost a quarter of the sample reported more than four fights in the past year, which could question the representativeness of the sample. Several studies, in fact, show a percentage ranging from 4 to 15%.^21^ Third, all measures used in this study were self-report tools, and future research on this topic should encompass observational measures, tools filled out by professionals and/or clinicians, or report form questionnaires, to increase the robustness of gathered data. Fourth, this study did not gather data on forms of violence and aggression other than physical fights (verbal aggression, for instance), nor did it consider whether youths started or were involved in the fight. Further, the participants of the study were not asked to report on emotional dysregulation in relation to the time when they engaged in fights. Actually, the ability to regulate emotions in general might be different from the ability to regulate emotions when triggered to fight. Future studies should address this issue. Finally, the self-reported fights could include fights among siblings (owing to the item construction of the questionnaire), which would not allow for a fair comparison between adolescents who have and do not have a sibling.

## Data Availability

The data that support the findings of this study are available from the corresponding author, L.C., upon reasonable request.
